# PEGylated versus Non-PEGylated pH-Sensitive Liposomes: New Insights from a Comparative Antitumor Activity Study

**DOI:** 10.3390/pharmaceutics14020272

**Published:** 2022-01-24

**Authors:** Shirleide Santos Nunes, Juliana de Oliveira Silva, Renata Salgado Fernandes, Sued Eustaquio Mendes Miranda, Elaine Amaral Leite, Marcelo Alexandre de Farias, Rodrigo Villares Portugal, Geovanni Dantas Cassali, Danyelle M. Townsend, Mônica Cristina Oliveira, André Luís Branco de Barros

**Affiliations:** 1 Department of Pharmaceutical Products, Faculty of Pharmacy, Universidade Federal de Minas Gerais, Av. Antônio Carlos, 6627, Belo Horizonte 31270-901, Brazil; shirleidenunes@yahoo.com.br (S.S.N.); julianaoliveira.far@gmail.com (J.d.O.S.); renatasalgadof@yahoo.com.br (R.S.F.); sued1989@gmail.com (S.E.M.M.); leite_elaine@hotmail.com (E.A.L.); itabra2001@yahoo.com.br (M.C.O.); 2 Brazilian Nanotechnology National Laboratory (LNNano), Brazilian Center for Research in Energy and Materials (CNPEM), Campinas 13083-100, Brazil; marcelo.farias@lnnano.cnpem.br (M.A.d.F.); rodrigo.portugal@lnnano.cnpem.br (R.V.P.); 3 Department of General Pathology, Institute of Biological Sciences, Universidade Federal de Minas Gerais, Av. Antônio Carlos, 6627, Belo Horizonte 31270-901, Brazil; geovanni.cassali@gmail.com; 4 Department of Drug Discovery and Pharmaceutical Sciences, Medical University of South Carolina, Charleston, SC 29425, USA; townsed@musc.edu; 5 Department of Clinical and Toxicological Analyses, Faculty of Pharmacy, Universidade Federal de Minas Gerais, Av. Antônio Carlos, 6627, Belo Horizonte 31270-901, Brazil

**Keywords:** liposomes, polyethylene glycol, antitumor activity, PEGylated liposomes, doxorubicin

## Abstract

PEGylated liposomes are largely studied as long-circulating drug delivery systems. Nevertheless, the addition of PEG can result in reduced interactions between liposomes and cells, hindering liposomal internalization into target cells. The presence of PEG on the surface of pH-sensitive liposomes is not advantageous in terms of biodistribution and tumor uptake, raising the question of whether the indiscriminate use of PEG benefits the formulation. In this study, two doxorubicin-loaded pH-sensitive liposomal formulations, PEGylated (Lip2000-DOX) or non-PEGylated (Lip-DOX), were prepared and characterized. Overall, the PEGylated and non-PEGylated liposomes showed no differences in size or morphology in Cryo-TEM image analysis. Specifically, DLS analysis showed a mean diameter of 140 nm, PDI lower than 0.2, and zeta potential close to neutrality. Both formulations showed an EP higher than 90%. With respect to drug delivery, Lip-DOX had better cellular uptake than Lip_2000_-DOX, suggesting that the presence of PEG reduced the amount of intracellular DOX accumulation. The antitumor activities of free-DOX and both liposomal formulations were evaluated in 4T1 breast tumor-bearing BALB/c mice. The results showed that Lip-DOX was more effective in controlling tumor growth than other groups, inhibiting tumor growth by 60.4%. Histological lung analysis confirmed that none of the animals in the Lip-DOX group had metastatic foci. These results support that pH-sensitive liposomes have interesting antitumor properties and may produce important outcomes without PEG.

## 1. Introduction

The key to successful anticancer therapies depends on the ability of the formulation to reach their target sites while minimizing accumulation and at non-specific tissues that attribute to side effects. The development of nanotechnology-based drug delivery systems (DDS) that can enhance the biodistribution, tissue uptake, and pharmacokinetics of therapeutic agents is a significant area in biomedical research. Among DDS, liposomes are a well-established platform that offers advantages such as biocompatibility, the capacity of self-assembly, high drug payload, in addition to the possibility of surface modification that can change their biological characteristics for different purposes [1,2].

Liposomal carriers have an impact on pharmacokinetics and tissue distribution of incorporated drugs, enhancing their therapeutic efficacy and reducing side effects [3]. PEGylated (Doxil®/Caelyx®) and non-pegylated (Myocet®) doxorubicin liposomes were the first in class approved, and today, they are utilized for the treatment of women with ovarian and breast cancer. Despite such advances, the successful use of DDS can be limited by several factors. Some limitations include the predominant uptake of liposomes by the mononuclear phagocyte system (MPS), the difficulty of predicting patterns of liposome extravasation, and long-term physicochemical stability are the main biological and chemical barriers that still need to be surpassed [4,5].

In recent decades, many strategies have been applied to overcome these challenges, including using the hydrophilic polyethylene glycol (PEG) polymer on the surface of the nanoparticles that serve to increase the circulation half-life and stability of formulations. This modification is one of the most well-established strategies for improving the effectiveness of therapeutic nanoparticles because it diminishes the recognition and clearance by MPS [6,7,8]. Consequently, the longer half-lives and slower clearance of PEGylated liposomes, also known as stealth or sterically stabilized liposomes, may result in therapeutic benefits [7,8,9]. Still, there are limitations associated with the addition of PEG. In some cases, the large chains from PEG on the surface of liposomes may reduce the interaction between liposomes and cell membranes, hindering internalization into the target cells [10,11,12]. In our previous study, we investigated the effect of PEG on biodistribution and tumor uptake of different types of pH-sensitive liposome formulations, and unexpectedly, the use of the polymer compromised the accumulation of the liposomes at the site of interest [10]. These findings questioned the value of indiscriminate use of PEG without a broad understanding of its benefit to the formulation as a whole. Therefore, the present study was designed to evaluate the influence of PEG on the antitumor activity of pH-sensitive liposomes in a 4T1 murine breast cancer tumor model.

## 2. Material and Methods

### 2.1. Material

The reagents and chemicals used in this study were of analytical grade and are commercially available. Specifically, Dioleoylphosphatidylethanolamine (DOPE, LIPOID PE 18:1/18:1), distearoylphosphatidylethanolamine (DSPE, LIPOID PE 18:0/18:0), and distearoylphosphatidylethanolamine polyethyleneglycol2000(DSPE-PEG2000, LIPOID PE 18:0/18:0–PEG2000) were supplied by Lipoid GmbH (Ludwigshafen, Germany). Cholesteryl hemisuccinate (CHEMS, Cat. # 850524P) was purchased from Sigma-Aldrich Chemical Company (St Louis, MO, USA). Doxorubicin hydrochloride (DOX, >98% purity) was purchased from ACIC Chemicals (Brantford, Ontario, Canada). D-(+)-Glucose (99,5% purity) was purchased from Vetec Química Fina Ltd.a (São Paulo, Brazil). The analytical reagents and solvents (high-performance liquid chromatography analytical grade) were purchased from Sigma-Aldrich (São Paulo, Brazil).

### 2.2. Liposome Preparation

PEGylated (Lip2000) and non-PEGylated (Lip) liposomal formulations were prepared as previously described [10,13]. Aliquots of lipids DOPE, CHEMS, and DSPE or DSPE-PEG2000 solubilized in chloroform (lipid concentration of 40 mM; molar ratio of 5.7:3.8:0.5, respectively) were transferred to a bottle, and the chloroform was removed under reduced pressure. A thin lipid film was formed and hydrated with a solution of NaOH (15 mM) and then with an ammonium sulfate solution (300 mM), at room temperature, under stirring. NaOH solution was used to guarantee complete ionization of CHEMS, which is indispensable to form vesicles, while ammonium sulfate was used to perform the DOX remote gradient encapsulation method. Then, vesicles were formed, and the final pH was adjusted to 7. The liposomes were extruded through 0.4 µm, 0.2 µm, and 0.1 µm polycarbonate membranes (5 cycles for each) to standardize the size. For DOX encapsulation, empty liposomes were incubated with 2 mg/mL of the drug, at 60 °C, for 1 h. Non-encapsulated DOX was removed by ultracentrifugation (2 h, 150,000× *g*, 4 °C).

### 2.3. Determination of Size Distribution, Zeta Potential, and Encapsulation Efficiency

Diameter, polydispersion index (PDI), and zeta potential were determined using NanoZS 90 Zetasizer (Malvern Instruments, Malvern, UK). Mean diameter and PDI was measured by dynamic light scattering at 25 °C at an angle of 90°, and zeta potential was determined by electrophoretic mobility determination at an angle of 90°. Samples were diluted in a NaCl 0.9% (w/v) solution, transferred to 12 mm square polystyrene cuvettes (mean diameter) or folded capillary zeta cells (zeta potential), and the measurements were performed in triplicate.

The DOX encapsulation percentage (EP) into liposomes was determined by high-performance liquid chromatography (HPLC) using a fluorescence detector (Waters Instruments, 1200 series, Milford, MA, USA) [14]. HPLC analyses were performed with a C8, 250 × 4.6 mm, 5-μm column (Merck, ACE^®^ 250-4.6, Aberdeen, Scotland), methanol: phosphate buffer 0.01 mol/L pH 3.0 65: 35 as the mobile phase, and flow rate of 1.0 mL/min. Samples (20 μL) were injected, and the eluate was monitored at excitation/emission wavelengths of 477/555 nm. For quantification of DOX in Lip and Lip2000, the lipid membrane was disassembled with isopropyl alcohol, and then the preparation was diluted appropriately in the mobile phase.

The EP was calculated according to the following Equation (1):

(1) $$EP=\frac{{\left[\mathrm{DOX}\right]}_{Purified\;Lip}\times 100}{{\left[\mathrm{DOX}\right]}_{Total\;Lip}}$$

where [DOX]*_Purified Lip_* are liposomes after the ultracentrifugation process described previously at Section 2.2 and contains only the encapsulated fraction of DOX and [DOX]*_Total Lip_* are the liposomes before ultracentrifugation.

### 2.4. Cryogenic Transmission Electron Microscopy

The samples were prepared in copper grids submitted to the glow discharge procedure with the following parameters: current of 15 mA; negative charge; 25 s of discharge. Freezing on amorphous ice using Vitrobot Mark IV (FEI, Netherlands) was performed at 22 °C and 100% moisture. Sample preparation followed the parameters: blot time 2.5 s; blot force −5, blot wait 20 s with a single blot. Then, 3 μL of the samples were applied to the grids and immersed in liquid ethane. After plunging, the grid was maintained in liquid nitrogen until the moment of analysis under the microscope and maintained at −173 °C throughout the microscopic analysis. Mean diameters were determined by measuring 100 nanoparticles for each sample using Image J software (version 1.53c).

### 2.5. Cell Culture

The murine breast cancer 4T1 cells were cultivated in RPMI 1640 medium (Gibco, Carlsbad, CA, USA), supplemented by 10% (v/v) fetal bovine serum (South America Origin—Gibco, USA) and 1% of PSA Antibiotic solution (penicillin, streptomycin, and amphotericin B) (Thermo Fisher Scientific—Sao Paulo, Brazil). Cells were maintained in sub-confluent growth conditions in a humidified incubator containing 5% CO_2_ at 37 °C. After 3–5 days of growth, the cells were removed from the flasks to perform cell uptake assays.

### 2.6. Cellular Uptake

The DOX uptake from Lip2000 and Lip were measured by HPLC, and internalization was confirmed by confocal microscopy. For HPLC analysis of internalization following dosage of DOX, 4T1 tumor cells were plated in 12-well plates (5 × 105 cells/well) and incubated for 24 h. Then, the cells were treated (n = 3) with 1 mL of fresh RPMI 1640 containing 3 µM of DOX, Lip2000-DOX, or Lip-DOX. The cells were incubated again for 1 and 4 h. The plates were washed with PBS at each time point to remove the drug, and cells were harvested from the plates using trypsin. After that, the cell suspensions were washed with PBS buffer twice using centrifugation (2000 rpm, 5 min) to remove the excess buffer. The resulting pellets were resuspended in 1 mL of methanol:isopropyl alcohol (1:1) solution, and the samples were transferred to the ultrasonic bath to promote cell lysis and DOX extraction. Finally, the suspension was centrifuged at 5000 rpm for 15 min, and the supernatant was used to determine DOX concentration by HPLC analysis as mentioned in Section 2.3. The DOX cellular uptake was calculated by Equation (2).

(2) $$cellular\;uptake\;\left(\%\right)=\frac{\left(\left[\mathrm{DOX}\right]extracted\;from\;cells\right)}{\left[\mathrm{DOX}\right]total}\;\times 100\;$$

The cells were plated in 6-well plates with sterile coverslips (2.5 × 105 cells/well) for confocal image analysis 24 h before treatment. Cells were incubated with 3 µM of DOX, Lip2000-DOX, or Lip-DOX for 4 h. Posteriorly, the treatments were removed, and the wells were washed with PBS buffer. Cells were fixed with formaldehyde 3.7% (v/v) solution and permeabilized with Triton X-100 0.1% (v/v) solution (FARACO et al., 2018). The coverslips were washed with PBS, and slides were assembled using Prolong Gold Antifade Reagent (Thermo Fisher Scientific—Waltham, MA, USA). The images obtained slides were analyzed in the Center for Image Acquisition and Processing at the Federal University of Minas Gerais (CAPI/UFMG—Belo Horizonte, Brazil) using the LSM 880 microscope with Airyscan detector (Zeiss—Oberkochen, Germany). The images were acquired using 40× objective lens, and Argonium 488 nm (excitation of DOX) laser was employed (SILVA et al., 2019). The images were processed using the ZEN Blue Edition software version 2.3 lite (Zeiss—Oberkochen, Germany).

### 2.7. In Vivo Antitumor Activity

Then, 8–10-week-old female BALB/c mice were purchased from CEBIO-UFMG (Belo Horizonte, Brazil). Animals were kept in a temperature- and humidity-controlled environment with free access to standard food and water. All experiments were conducted under the approval of the Ethics Committee on Animal Use (CEUA) of the Federal University of Minas Gerais, Brazil (Protocol # 134/2018).

To enable the proper growth of the tumor, cells were maintained for 10 days for in vivo analysis. Aliquots (100 µL) containing 1.0 × 106 4T1 cells were subcutaneously injected into the right flank of female BALB/c mice. After tumor development, mice were randomly separated into five treatment groups (n = 7 for each group): (1) Blank non-PEGylated liposomes (Lip-control group); (2) Blank PEGylated liposomes (Lip2000-control group); (3) free DOX; (4) DOX-loaded non-PEGylated liposome (Lip-DOX); and (5) DOX-loaded PEGylated liposome (Lip2000-DOX). The cumulative dose of DOX in all treatment groups was 16 mg/kg, separated into 4 administrations (4 mg/kg), every 3 days. The mice were weighed throughout the study, and tumors were measured with a caliper every 2 days until the end of the experiment (D14), when mice were sacrificed. Tumor volumes (TV) were calculated by the formula (3):

TV = (d_1_)^2^ × d_2_ × 0.5 (3)

where d_1_ and d_2_ represent the smallest and largest diameter, respectively, the relative tumor volume (RTV) and the inhibition ratio (IR) were calculated on D14, as follows:

(4) $$RTV=\frac{TV\;on\;D14}{TV\;on\;D0}\;\;\;\;\;\;\;\;$$

(5) $$IR\left(\%\right)=1-\frac{Mean\;RTV\;of\;drug-treated\;group}{Mean\;RTV\;of\;control\;group}\times 100\;\;\;\;\;\;$$

### 2.8. Histological Analysis

Following sacrifice, the tumor, liver, kidneys, lungs, spleen, and heart tissues were collected and processed for histopathological analysis. The tissues were fixed in 10% buffered formalin for 48 h, dehydrated in alcohol, and included in paraffin blocks. Sections of 4 μm were obtained and stained with hematoxylin and eosin (H&E). A trained pathologist analyzed the slides, and afterward, the images were captured by a camera connected to an optical microscope (Olympus BX-40; Olympus, Tokyo, Japan).

### 2.9. Statistical Analysis

Data were expressed as the mean ± standard deviation (SD). Differences between the experimental groups were assessed using one-way ANOVA analysis of variance followed by Tukey’s test. The 95% confidence interval was adopted for all analyses, and the differences were considered significant when the p-value was <0.05. Data were evaluated with GraphPad Prism software (version 5.00, La Jolla, CA, USA).

## 3. Results

### 3.1. Liposomes Physicochemical Characterization

The mean diameter, polydispersity index (PDI), zeta potential, and EP of the liposomal formulations are summarized in Table 1.

Consistency between the formulations was validated. Specifically, all formulations showed a similar mean diameter with an average particle size smaller than 200 nm. Encapsulation of DOX into either liposome did not affect the mean size. A PDI value <0.3 indicates that the particles are evenly distributed in the system. The zeta potential was essentially neutral for the two liposomes. Lip showed a more negative zeta potential value since there is no PEG chain on the surface hiding the negative charges of the structural lipids that compose the nanoparticle. The value of EP was >90% for all formulations (93.1 ± 1.2% for the Lip, and 92.3 ± 0.9% for Lip2000). These results indicate that the method of encapsulation of DOX in liposomes by ammonium sulfate gradient was equivalent.

### 3.2. Cryogenic Transmission Electron Microscopy

**Figure 1:**
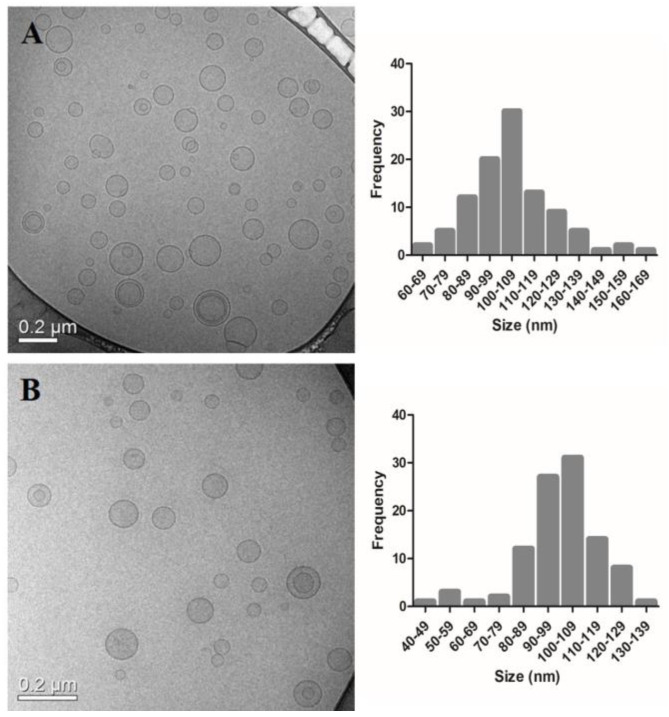
Cryogenic Transmission electron microscopy images and mean diameter distribution of Lip (**A**) and Lip_2000_ (**B**). Round shape vesicles with a similar size distribution are observed, which is of pivotal importance for comparative purposes.

The images obtained by cryogenic transmission electron microscopy (cryo-TEM) for the blank liposomes (Figure 1) allowed the visualization of unilamellar vesicles with an average diameter around 100 nm. Still, liposomes in the range of 30–250 nm were also observed. No differences were found in size and the morphology between PEGylated and non- PEGylated liposomes. Cryo-TEM images were only made for the blank formulations since the encapsulation of DOX did not impair the mean diameter measured by DLS. The analysis of vesicle size by cryo-TEM corroborates the results found by DLS. Each technique showed similar sizes for the formulations, as it was possible to visualize monodisperse populations.

### 3.3. Cellular Uptake

**Figure 2:**
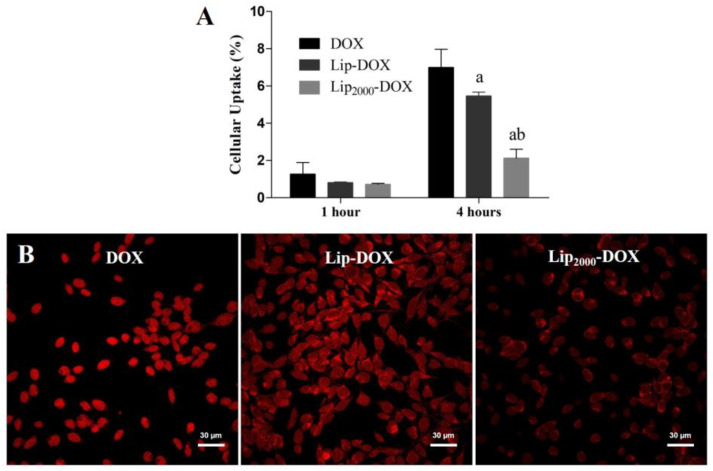
Cellular uptake of DOX by HPLC analysis after 1 h and 4 h incubation with DOX, Lip-DOX, and Lip_2000_-DOX (**A**) and cellular uptake of DOX by confocal microscopy images after 4 h of incubation with DOX, Lip-DOX, and Lip_2000_-DOX (**B**). Data are expressed as the mean (n = 3) ± standard deviation. All data were analyzed by one-way ANOVA analysis of variance followed by Tukey’s post-test. ^a^ Represents statistical differences (*p* < 0.05) between the DOX and Lip-DOX or Lip_2000_-DOX. ^b^ Represents statistical differences (*p* < 0.05) between the Lip-DOX and Lip_2000_-DOX.

The cellular uptake of DOX, Lip-DOX, and Lip2000-DOX is demonstrated in Figure 2. Importantly, Lip-DOX had higher cellular uptake than Lip2000-DOX at 4 h post-incubation (*p* < 0.05), which suggests that the presence of PEG reduces the access of the drug to the interest cells (Figure 2A). Supporting these data, similar uptake behavior was observed in confocal image analysis (Figure 2B). Cells treated with DOX and Lip-DOX, in qualitative analysis, showed higher intracellular fluorescence than cells treated with Lip2000-DOX (*p* < 0.05).

### 3.4. Antitumor Activity

**Figure 3:**
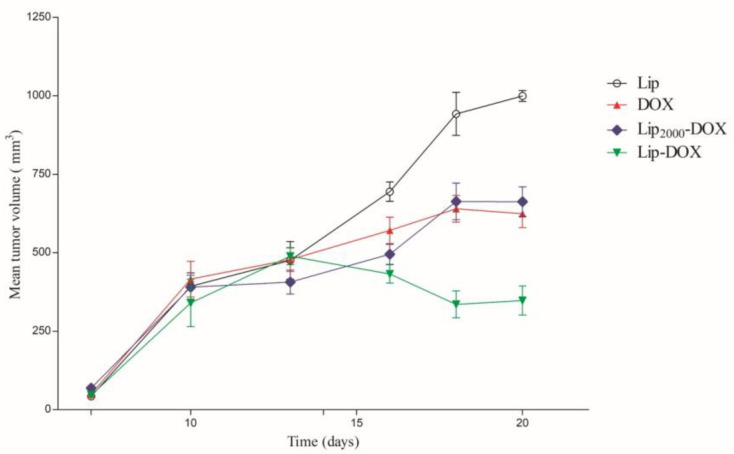
Antitumor effects of free DOX, blank-liposome (Lip), Lip-DOX, and Lip_2000_-DOX on the growth of 4T1 breast cancer cells subcutaneously implanted in BALB/c female mice (n = 6). The treatments were intravenously administered 4-times, every three days, at a 4 mg/kg dose. The total dose in all DOX-treated groups was 16 mg/kg.

The antitumor activity of free DOX and liposomes was evaluated in the 4T1 tumor model in BALB/c mice. Tumor volume over time is shown in Figure 3. Both control groups (Lip and Lip2000) showed identical tumor growth profiles; therefore, only Lip is represented in Figure 3.

All animals treated with DOX, independent of formulation, showed a favorable tumor growth relative to the control treatment group, Figure 2. These data are confirmed using an alternative fitted model (Table 2). Importantly, the tumor volume of the Lip-DOX treated mice was significantly decreased after 15 days relative to the DOX or Lip2000-DOX treated animals. In contrast, no statistical differences were observed between Lip2000-DOX and free DOX treated animals. Additionally, the inhibition ratio (IR) corroborates the data found by tumor volume measurements, Table 2. The Lip-DOX treatment group inhibited tumor growth by 60.4%, whereas free DOX and Lip2000-DOX treatment inhibited tumor growth by only 36.6% and 40.3%, respectively.

### 3.5. Histological Analysis

**Figure 4:**
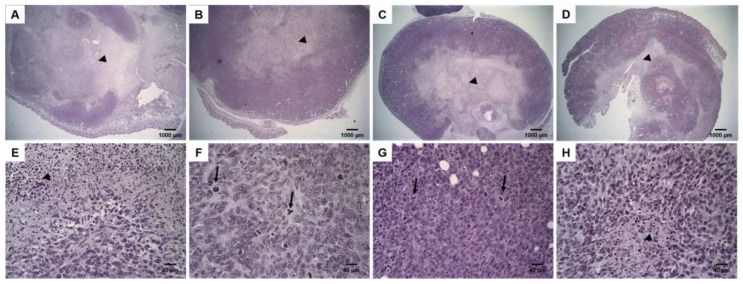
Histological sections of primary tumor from 4T1 breast tumor-bearing female BALB/c mice. Control (**A**,**E**); free-DOX (**B**,**F**); Lip-DOX (**C**,**G**); Lip_2000_-DOX (**D**,**H**) stained by hematoxylin and eosin. The black arrowheads indicate the necrosis areas. The black arrows indicate mitosis figures—magnification 2× and 40×.

**Figure 5:**
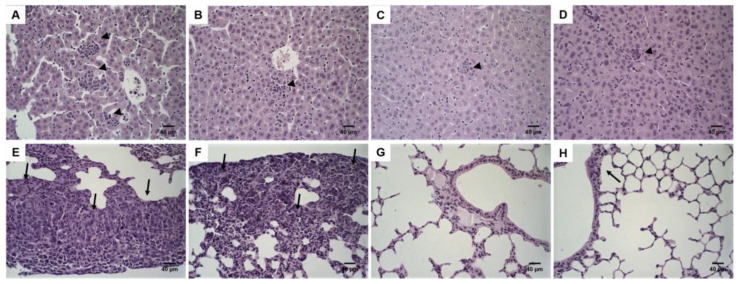
Histological sections of liver and lungs from 4T1 breast tumor-bearing female BALB/c mice. Control liver (**A**); free-DOX liver (**B**); Lip-DOX liver (**C**); Lip_2000_-DOX liver (**D**) stained by hematoxylin and eosin. Control lungs (**E**); free-DOX lungs (**F**); Lip-DOX lungs (**G**); Lip_2000_-DOX lungs (**H**) stained by hematoxylin and eosin. The black arrowheads indicate inflammation. The black arrows indicate metastasis foci. No metastatic focus was observed in the lungs of Lip-DOX-treated mice—magnification 40×.

DOX treatment induces tumor necrosis. Figure 4 shows histological sections of tumor tissues. Solid tumors with a central necrosis area were observed for all treatment groups (Control, free-DOX, Lip-DOX, and Lip_2000_-DOX). The tumor cells were characterized by round nuclei, broad cytoplasm, and elevated pleomorphism. Typical and atypical mitoses were observed in all groups. These morphological features are compatible with 4T1 cells murine breast carcinoma [15], also characterized by aggressive growth and metastatic behavior, with lung metastases around 14 days after the inoculum [16]. The 4T1 breast cancer model shows metastasis to the lungs, similar to that in human patients. Histological sections of the lungs from each treatment group were evaluated. The analysis showed some metastatic foci, characterized by groups of epithelial cells among the alveoli, in one animal per group of control, free-DOX, and Lip_2000_-DOX groups. Noteworthy, none of the animals in the Lip-DOX group showed metastatic foci (Figure 5E–H). These data suggest that the Lip-DOX-treated group controlled metastatic progression by preventing and/or delaying the tumor cell spreading. In the liver analysis, inflammatory foci were observed. Mice from the control group showed intense multifocal inflammatory infiltrate; meanwhile, in the treated groups, this effect was decreased (Figure 5A–D). In the spleen, tissue hyperplasia was found for all groups. However, this aspect was equally attenuated in mice that received free-DOX, Lip-DOX, and Lip_2000_-DOX [17] (Figure S1).

**Figure 6:**
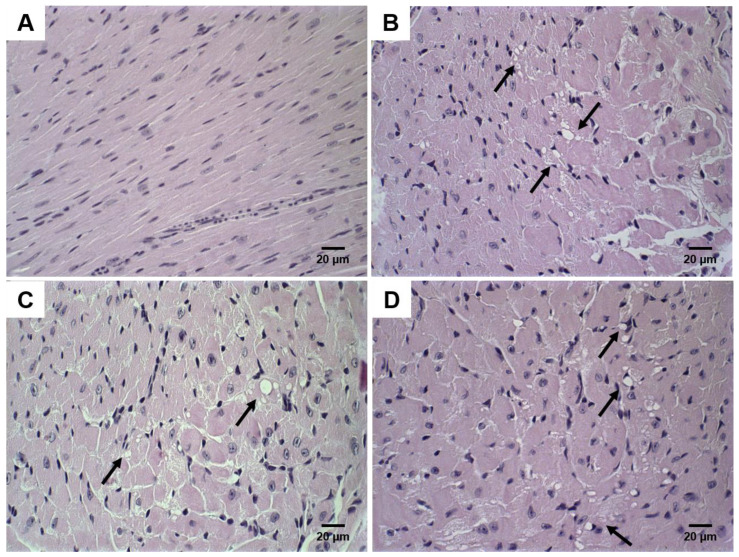
Histological sections of heart from 4T1 breast tumor-bearing female BALB/c mice. Control (**A**); free-DOX (**B**); Lip-DOX (**C**); Lip_2000_-DOX (**D**) stained by hematoxylin and eosin. The black arrows show vacuolization areas, compatibles with heart cell degeneration—magnification 60×.

Histological sections of the heart and kidney were also analyzed in order to identify DOX-related toxic effects. Morphological changes in heart tissue are common in chemotherapy regimens using DOX. Discrete and focal heart degeneration was observed in groups treated with chemotherapy. The degeneration was characterized by vacuolization in cardiomyocytes’ cytoplasm (Figure 6).

## 4. Discussion

Predicting an encapsulated drug’s in vivo behavior and therapeutic performance is a major challenge in developing nanoparticle-based DDS for cancer treatment. In this context, advances in applying drug-loaded conventional and PEGylated liposomes have been achieved, leading to improved pharmaceutical efficacy over free drugs in certain tumor models [1,4]. However, several studies have shown that differences in liposome composition dramatically alter the pharmacokinetics, pharmacodynamics, and cellular uptake. For a drug to be bioactive, it must reach the target site at the appropriate concentration and, most importantly, become incorporated into the target cells. As discussed previously, the modification of the surface of the liposomes with hydrophilic molecules, such as PEG, increases the blood circulation retention time of nanoparticles by reducing their uptake by MPS [18]. While this leads to a more favorable pharmacokinetic parameter, the surface modification may reduce the uptake of the nanoparticles by the cells of interest [19]. Consequently, the changes in circulation time may not enhance antitumor activity.

In a previous study, the pharmacokinetics and biodistribution of conventional and PEGylated pH-sensitive liposomes were evaluated, and results showed similar clearance and high tumor accumulation for both liposomes regardless of the presence of PEG, indicating that the presence of the hydrophilic layer did not show beneficial effects in the in vivo properties of these pH-sensitive liposomes [10]. The present study analyzed how this non-PEGylated formulation performed in extended antitumor activity assays. All liposome formulations (PEGylated or not) were designed and prepared to achieve similar physicochemical properties. The size of the diameters of the particles should be <200 nm to facilitate evasion by the MPS and ensure incorporation through the membrane of target cells [20]. The nanoparticles developed herein were consistent and differed by whether they were PEGylated or not. This is supported in both cryo- -microscopy and the DLS data. Specifically, the Zeta potential values close to neutrality were found for each liposomal preparation. As expected, Lip presented a negatively charged zeta potential, probably due to the absence of PEG. Without the PEG-coating, negative charges from the structural lipids might be exposed, leading to more negative zeta potential than PEGylated liposomes [21]. Furthermore, the addition of PEG onto the nanoparticle surfaces can reduce the electrophoretic mobility of the vesicle, resulting in a zeta potential value for Lip2000 closer to neutrality than the non-PEGylated system (Lip) [21]. DOX was efficiently encapsulated, via ammonium gradients, in both liposomes, and the incorporation of the drug did not affect the physicochemical aspects of the liposomes, facilitating continued comparative in vitro and in vivo studies.

The role of PEGylation on cellular impact and, ultimately, antitumor activity was explored. We showed that a significantly higher cellular uptake was achieved for Lip-DOX in comparison to Lip2000-DOX. These data combined with more favorable in vivo growth inhibition support our hypothesis that PEG may impair the internalization of the drug within the cell both in vitro and in vivo. The PEGylated liposomes reached cellular uptake homeostasis with significantly inferior times to Lip-DOX. Wang and co-workers reported that several factors might interfere with the ability of formulations to exert their desired action [22]. In this context, PEG assumes a pivotal influence since, despite its importance for nanocolloidal stability and extended circulation half-life, it might significantly reduce internalization by cells, including tumors, as a result of the hydrophilic corona and high steric hindrance conferred by this polymer. Studies performed by Verhoef and Anchordoquyin, 2013, showed similar results concerning the use of the PEG polymer [5]. The addition of PEG onto the liposomes’ surface was proposed to decrease macrophage uptake, responsible for prolonging blood circulation time in vivo [23,24,25]. However, at the same time, the steric hindrance that decreases the interaction with the SFM may prejudice the interaction with the tumor cells, consequently compromising the internalization [26].

While endocytosis is implicated in the drug delivery mechanism by liposomes, this may not be the whole story. For example, liposomes are adsorbed onto the cell surface and subsequently internalized [27], and the efficient liposome internalization and subsequent release of encapsulated drugs can explain enhanced liposome pharmacological activity. The presence of the bulky PEG chains could reduce the cell internalization of PEGylated liposomes [19,28]. Despite the aforementioned changes, the absence of PEG possibly did not affect the pH sensitivity of the formulation. The naturally low pH of endosomes (~pH 5.0) triggers DOX release from the liposomes. The presence of DOPE in the liposome bilayer is one determinant of this pH responsiveness. DOPE molecules are hexagonally organized instead of lamellar structures in an aqueous medium. CHEMS, a carboxylated lipid, is used as a stabilization agent for DOPE. At physiological pH, ionization of the CHEMS inserted between the DOPE molecules occurs, promoting an electrostatic repulsion between the CHEMS ionized carboxyl and the DOPE phosphate groups. Repulsion between these groups allows the lamellar organization, and consequently, spontaneous liposomal formation. Therefore the lower pH in the endosomal results in the protonation of CHEMS molecules, and consequently, destabilization of the vesicles encourages drug release [29].

The in vivo antitumor experiments corroborated the in vitro findings. Lip-DOX was more effective in controlling tumor growth, while Lip2000-DOX and the free drug exhibited similar profiles but less efficient than non-PEGylated liposomes. Indeed, IR values showed an improvement by at least 50% when Lip-DOX was used in contrast to the other treatments. These findings support our previous study in which, for this specific formulation, PEG did not improve pharmacokinetic parameters, and a similar biodistribution, including tumor uptake, was observed for non-PEGylated and PEGylated liposomes [10]. In this scenario, both formulations can reach the tumor site at the same rate, and PEG will contribute negatively to the internalization of the vesicles and, therefore, to antitumor activity, as confirmed by our in vivo results. Other authors have reported the limitations of the presence of PEG moieties in drug delivery since the polymer might reduce the interaction between the lipid bilayer of the vesicles and the tumor cell membrane, leading to reduced drug delivery and, consequently, decreased antitumor activities [30]. The importance of PEG in the stability and increase in the circulation time of nanoparticles is already widely supported. However, it depends on the concentration and size of the polymer, which, in turn, can change its shape and interact with other structures. Therefore, the effects of the addition of this polymer to the surface of the particle must be carefully evaluated to achieve the desired action [18].

4T1 tumor is a well-established primary and metastatic breast tumor model that allows a single assay to evaluate the efficacy. Lung metastases occurred in animals from all treatment groups, except the Lip-DOX formulation. As reported in other studies, DDS probably acts in preventing and/or delaying the dissemination of viable cells from the primary tumor. Micrometastases may be relatively avascular, being harder to target with nanocarriers; therefore, the drug may not have direct cytotoxic effects [28,29]. Additionally, no significant sign of toxicity was observed in the evaluated tissues indicating low side effects of Lip-DOX.

## 5. Conclusions

In this study, DOX-loaded PEGylated and non-PEGylated liposomes were efficiently prepared with similar mean diameters, size distributions, and zeta potentials along with high drug entrapment efficiencies (>90%). The results of our antitumor activity studies showed that Lip-DOX was more efficient in controlling tumor growth, indicating that PEG did not improve in vivo properties of the pH-sensitive liposomes used in this study, questioning the utility of PEG in liposomal formulations. Critical then is to evaluate the balance between employing PEG at higher levels in attempts to enhance tumor accumulation via EPR and using PEG in a minimal amount to ensure maximum uptake/delivery to target tissues.

## Figures and Tables

**Table 1:** Mean Diameter, PDI, zeta potential, and EP of liposomal formulations. The results were expressed as mean ± standard deviation (n = 3). ^a^ Represents a significant difference between Lip and Lip_2000_ (*p* < 0.05). ^b^ Represents significant difference between Lip-DOX and Lip_2000_-DOX (*p* < 0.05).

Formulation	Mean Diameter (nm)	PDI	Zeta Potential (mV)	EP
Lip	136.3 ± 3.9	0.12 ± 0.09	−9.7 ± 1.2	-
Lip_2000_	137.0 ± 2.1	0.13 ± 0.07	−4.1 ± 0.6 ^a^	-
Lip-DOX	139.4 ± 3.8	0.11 ± 0.07	−8.9 ± 0.8	93.1 ± 1.2
Lip_2000_-DOX	140.2 ± 3.1	0.12 ± 0.08	−4.0 ± 1.2 ^b^	92.3 ± 0.9

**Table 2:** Regression analysis of data for antitumor activities and values for tumor growth inhibition ratios (IR) after administration of blank-liposome (Lip), free DOX, Lip-DOX, and Lip_2000_-DOX.

**Treatment**	**Best-Fit Model**	**Correlation Coefficient (r^2^)**	IR (%)
Lip	y = 72.2x − 419.7	0.9723	-
DOX	y = −4.9x^2^ − 176.5x − 917.3	0.9675	36.1
Lip_2000_-DOX	y = −1.9x^2^ − 93.3x − 450.7	0.9238	40.3
Lip-DOX	y = 0.4x^3^ − 24.8x^2^ − 426.7x − 1886	0.9916	60.4

## Data Availability

Not applicable.

## Supplementary Materials

The following are available online at https://www.mdpi.com/article/10.3390/pharmaceutics14020272/s1, Figure S1: Histological sections of spleen from 4T1 breast tumor-bearing female BALB/c mice. Control (A); Lip-DOX (B) stained by hematoxylin & eosin. Amplification of 5×.
