# Supplementary material for: PEGylated versus Non-PEGylated pH-Sensitive Liposomes: New Insights from a Comparative Antitumor Activity Study
Source: Pharmaceutics. 2022 Jan 24;14(2):272. doi: 10.3390/pharmaceutics14020272 (PMC8874560; doi:10.3390/pharmaceutics14020272)
Supplement: Supplementary file 1 [file pharmaceutics-14-00272-s001.zip › pharmaceutics-1537842-supplementary.pdf]

pharmaceutics

MDPI

PEGylated *versus* non-PEGylated pH-sensitive liposomes: New insights from a comparative antitumor activity study

Shirleide Santos Nunes, Juliana de Oliveira Silva, Renata Salgado Fernandes, Sued Eustaquio Mendes Miranda, Elaine Amaral Leite, Marcelo Alexandre de Farias, Rodrigo Villares Portugal, Geovanni Dantas Cassali, Danyelle M. Townsend, Mônica Cristina Oliveira and André Luís Branco de Barros

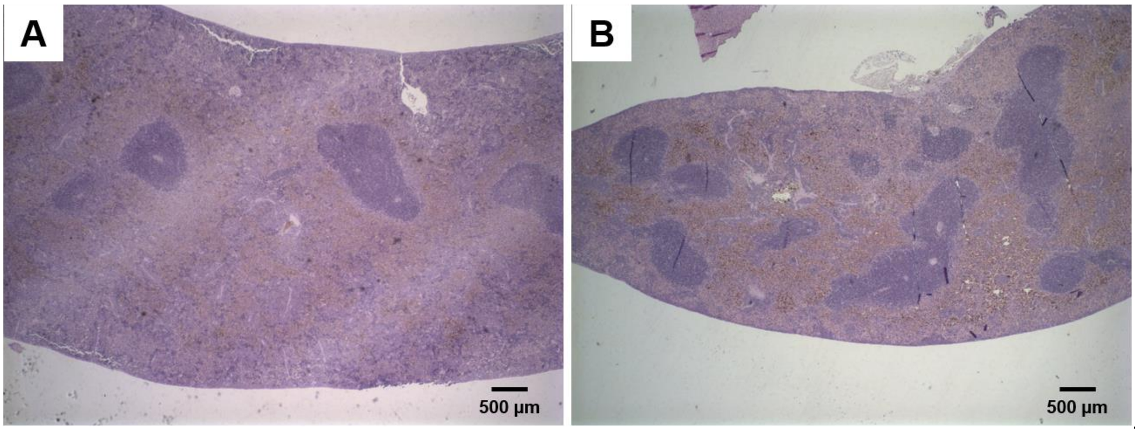

Figure S1. Histological sections of spleen from 4T1 breast tumor-bearing female BALB/c mice. Control (A); Lip-DOX (B) stained by hematoxylin & eosin. Amplification of 5x.
